# Lying Down Nystagmus in Lateral Canal Paroxysmal Positional Vertigo

**DOI:** 10.3390/audiolres16010008

**Published:** 2026-01-08

**Authors:** Mauro Gufoni, Nicola Ducci, Davide Bernacca, Luigi Califano, Augusto Pietro Casani

**Affiliations:** 1 Department of Otorhinolaryngology, Pisa University Hospital, 56122 Pisa, Italy; mgufoni@gmail.com (M.G.); 29550320@studenti.unipi.it (N.D.); davide.bernacca@gmail.com (D.B.); 2 Department of Audiology and Phoniatrics, San Pio Hospital, 82100 Benevento, Italy; luigi.califano1958@gmail.com

**Keywords:** geotropic, apogeotropic nystagmus, paroxysmal positional vertigo, canal jam, canalolithiasis, cupulolithiasis

## Abstract

**Introduction**: The aim of this study was to determine the position of otoconial debris in lateral ampullar or non-ampullar canalolithiasis, based on two parameters: (1) the direction of the nystagmus appearing when the patient lies down, if present, and (2) the positional nystagmus evoked by the supine roll test. **Methods**: Theoretical results were compared with a population of 170 patients observed over the past ten years for horizontal canal benign paroxysmal positional vertigo (HC-BPPV). The series included 141 geotropic and 29 apogeotropic cases. **Results**: Among the geotropic forms, 80 showed no supine nystagmus (Geotropic Nystagmus with no supine nystagmus, GT0) (56.7%), 51 had supine nystagmus directed toward the healthy side (Geotropic Nystagmus with supine nystagmus congruent, direct toward the healthy side, GT+) (36.2%), and 10 toward the affected side (Geotropic Nystagmus with supine nystagmus incongruent direct to the affected side, GT−) (7.1%). In the apogeotropic group, 10 showed no supine nystagmus (Apogeotropic nystagmus with no supine nystagmus, AGT0) (34.6%), 16 had nystagmus toward the affected side (Apogeotropic Nystagmus with supine nystagmus congruent, direct toward the affected side, AGT+) (55.2%), and 1 toward the healthy side (Apogeotropic Nystagmus with supine nystagmus Incongruent, direct toward the healthy side, AGT−) (3.4%). Two cases presented monopositional apogeotropic nystagmus (mAGT), consistent with a “sieve-type canal jam” (6.8%). Overall, 90 out of 170 patients (52.9%) showed no nystagmus in the supine position, with a statistically significant difference between variants (*p* = 0.0474, Yates correction). **Conclusions**: The comparison between lying-down nystagmus and positional nystagmus, assessed through the Supine Roll Test as the leading diagnostic maneuver for horizontal canal involvement, may help identify the initial location of debris within the lateral semicircular canal and guide the appropriate liberatory maneuver, while the effectiveness and side of the maneuver allow the distinction between canal-side and utricular-side jams.

## 1. Introduction

Most authors today agree that canalolithiasis originates from otolithic debris that migrates into a semicircular canal, where it moves freely, encountering resistance only from the endolymph and the canal walls. Accordingly, head movement along the appropriate plane can displace these fragments, generating endolymphatic currents that elicit transient, paroxysmal nystagmus [1,2,3].

In contrast, the pathogenesis of cupulolithiasis—where debris adheres to the cupula, making it responsive to gravity—remains controversial. Schuknecht’s original hypothesis [4] proposed that debris adheres directly to the cupula, but this has been questioned [5], as experimental studies suggest the otoconial fragments do not possess adhesive properties of that magnitude [6].

With the acceptance that otoconial debris may form a “jam,” a new question arises: could the phenomenon known as “cupulolithiasis” instead result from a mobile jam [5]? Paroxysmal positional vertigo involving the horizontal semicircular canal (HSC-PPV) encompasses a spectrum of nystagmic patterns that reflect distinct pathophysiological configurations of otolithic debris within the canal. The geotropic form, which represents the most frequent clinical presentation, is generally attributed to canalithiasis with free-floating particles gravitating toward the posterior arm of the canal, where head roll movements induce an ampullopetal endolymphatic flow consistent with Ewald’s second law.

A second group of cases displays a bilateral apogeotropic nystagmus that may spontaneously convert into a geotropic pattern or do so following appropriate positional maneuvers. This transitional behavior is typically interpreted as evidence of otoconial aggregates initially located in the anterior arm of the canal, where their position generates an ampullofugal deflection of the cupula. Subsequent migration of the debris toward the posterior arm leads to the emergence of the classical geotropic variant, indicating that this configuration represents an unstable or intermediate stage within the canalithiasis spectrum.

Conversely, a persistent apogeotropic nystagmus that does not evolve into a geotropic form is generally regarded as the clinical expression of a stable cupulolithiasis. In this scenario, the otolithic material is presumed to be located within or adjacent to the ampulla. Following head movements, this mass may exert a sustained deflecting force on the cupula or, alternatively, may obstruct the canal lumen near its junction with the cupula, thereby generating a persistent suction phenomenon. Both mechanisms induce a constant ampullofugal bias, producing a durable apogeotropic nystagmus that aligns with the classical features described for cupulolithiasis.

The concept of lying-down nystagmus (LDN) was first characterized as a reliable lateralizing sign in horizontal canal BPPV and later confirmed with evidence highlighting its correlation with otoconial movement and canal orientation [7,8,9,10].

In this manuscript, the nystagmus that appears when the patient lies down will be identified with the term “lying down nystagmus” or “supine nystagmus”, expressions already present in the literature.

More recent studies have provided further insights into the dynamics of horizontal and vertical components of nystagmus during the supine roll test, as well as the coexistence of direction-changing responses [11,12].

While this hypothesis is compelling, it remains unproven. It is also plausible that both mechanisms—true cupulolithiasis and mobile jams—exist independently.

Finally, recent therapeutic contributions demonstrated that the body-roll test and shortened forced positions can optimize both the diagnosis and management of lateral canal variants [13,14].

The mobile jam theory offers a valuable framework to interpret non-paroxysmal positional vertigo. In this study, we aim—focusing exclusively on the horizontal canal—to evaluate the expected clinical signs depending on the topographic location of the debris within the canal. This approach yields purely theoretical predictions that can be compared against clinical data.

## 2. Materials and Methods

**Figure 1:**
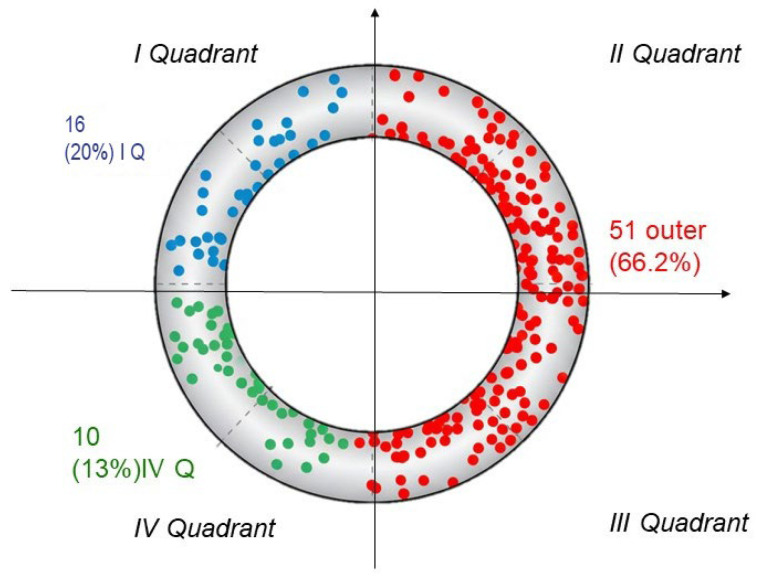
Schematic representation of the lateral semicircular canal seen from above. GT+ (red), GT− (green), and AGT+ (blue) cases are shown individually. The visual impression of a greater concentration of cases in the external area of the canal has been shown to be statistically significant. One reason for the asymmetry may be that natural rotational movements of the head induce centrifugal acceleration that displaces the debris.

We considered the lateral semicircular canal (findings are symmetrical for the contralateral side), visualized from above on a horizontal plane. The canal was divided into four quadrants using orthogonal Cartesian axes (Figure 1):

1. Quadrant I: includes the ampulla and the ascending segment of the canal (positive X), up to its highest point;
2. Quadrant II: negative X and positive Y;
3. Quadrant III: negative X and negative Y;
4. Quadrant IV: positive X and negative Y, corresponding to the non-ampullary arm’s entry into the utricle.

We assessed the effects of debris in each quadrant during key semiological maneuvers: (a) transitioning to the supine position (clinostatism), (b) head rotated to the right or left while supine.

This approach assumes that the patient is initially examined while sitting to assess for spontaneous nystagmus. The transition to a supine position is then evaluated for the onset, disappearance, or change in nystagmus.

We recorded the presence and direction of expected nystagmus based on the debris location at time t = 0.

To validate theoretical predictions, we analyzed a homogeneous cohort of patients diagnosed with either geotropic or apogeotropic lateral canalolithiasis between 2009 and 2018. Patients exhibiting central signs of vestibular pathology were excluded from the study.

All patients underwent a comprehensive assessment, including history, otoscopy, audiometry, spontaneous and evoked nystagmus testing (ocular and head-based, more specifically, the head-shaking test, HST and Skull Vibration–Induced Nystagmus SVIN), and positional maneuvers. All diagnostic maneuvers were recorded using video-oculography, which ensured complete removal of visual fixation throughout the examination. Positional nystagmus was evaluated using the Dix-Hallpike test [15] to rule out posterior canal BPPV, followed, when posterior canal involvement was excluded, by the Supine Roll Test (Pagnini-McClure maneuver) [3] to assess horizontal canal involvement. Additional assessments included the Head Shaking Test, vestibulospinal reflexes, and caloric testing (Fitzgerald-Hallpike) with slow-phase velocity measured using Jongkees’ formulas and recorded via video-oculography.

The final study population comprised 170 patients (95 females, 75 males; mean age 63 years, range 15–93).

Patients diagnosed with lateral canalolithiasis were treated with the Gufoni maneuver—on the affected side for apogeotropic forms, and on the healthy side for geotropic forms—followed by postural advice according to Vannucchi [16].

## 3. Results

Findings for the 170 patients are summarized in Table 1 and Table 2. Among them, 141 cases were classified as geotropic, and 29 as apogeotropic.

Of the geotropic cases:

- 80 patients (56.7%) showed no nystagmus upon assuming the supine position but exhibited positional nystagmus when the head was rotated laterally (GT0).
- 51 patients (36.2%) had nystagmus directed toward the healthy side when supine (congruent nystagmus, GT+).
- 10 patients (7.1%) had nystagmus directed toward the affected side (incongruent nystagmus, GT−).

Of the apogeotropic cases:

- 10 patients (34.5%) showed no nystagmus at clinostatism but presented with positional nystagmus during lateral head rotation (AGT0).
- 16 patients (55.2%) had nystagmus directed toward the affected side (AGT+, congruent).
- 1 patient (3.4%) had nystagmus directed toward the healthy side (AGT−, incongruent).
- 2 patients (6.9%) exhibited monopositional apogeotropic nystagmus (mAGT).

In total, 90 out of 170 patients (52.9%) did not exhibit nystagmus upon assuming the supine position. This occurred more frequently in geotropic cases (80/141, 56.7%) than in apogeotropic cases (10/29, 34.5%). This difference is statistically significant (Yates-corrected *p* = 0.0474), suggesting that lying-down nystagmus is more common in apogeotropic variants.

To demonstrate that the distribution is not due to chance and that it is significantly skewed, as suggested by centrifugal acceleration theory, a statistical goodness-of-fit test is needed. The most appropriate test in this case is the Chi-Square Test.

1. The Null Hypothesis H0: Assuming that the four quadrants have a geometrically equivalent area: Expected Probability for each Quadrant: P_expected = 1/4 = 0.25 (25%).
2. Expected Frequencies (E). If the distribution were random and uniform, with *n* = 77 total cases, the expected number of cases in each quadrant would be: E{I} = E{II} = E{III} = E{IV} = 77 × 0.25 = 19.25.
  For model grouping (Outer vs. Inside):
  External Expected Frequency (EEF_Outer): E{II} + E{III} = 19.25 + 19.25 = 38.5.
  Internal Expected Frequency (IEF_Inner): E{I} + E{IV} = 19.25 + 19.25 = 38.5.
3. Observed Data (O). We group data into two categories to maximize the power of the test against hypothesis:
  External Observed Frequency (EOF: O{II} + O{III} = 51.
  Internal Observed Frequency (IOF) = 16 + 10 = 26.
4. Calculation of the Chi-Square Test.
  Applying the formula to the two groupings (External and Internal): Chi-squared = 8.116 (1 degree of freedom, df).
  Critical Value: for df = 1 and a standard significance level of alpha = 0.05 (which equates to a 95% probability), the critical value of Chi-squared is 3.841. Since calculated Chi-squared value (8.116) is greater than the critical value (3.841), we reject the null hypothesis (H0).
5. Statistical Conclusion.
  The result shows that the probability of observing such an unbalanced distribution (51 vs. 26) if the true probability were uniform (38.5 vs. 38.5) is very low. In practical terms, the distribution of fragments is not due to randomness and is significantly skewed towards the outer quadrants, supporting hypothesis that a factor such as centrifugal acceleration (or another non-random mechanism) is at play.

## 4. Discussion

**Figure 2:**
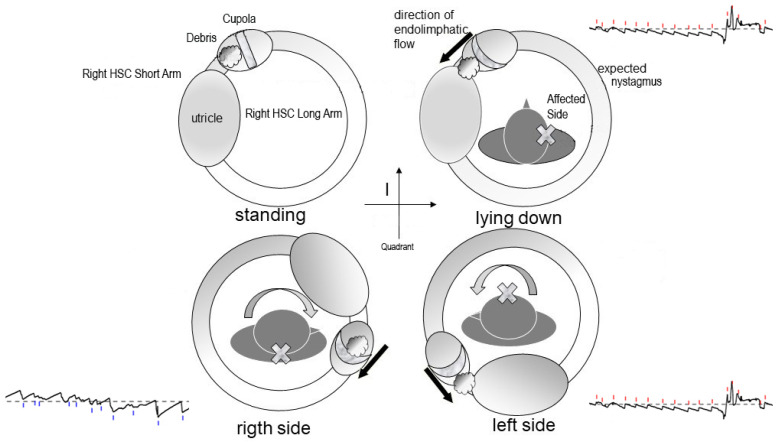
Schematic representation of otoconial debris located near the ampulla in the right horizontal semicircular canal and its expected movement during upright, supine, and lateral head positions. The resulting endolymphatic flow directions generate the corresponding horizontal nystagmus patterns shown in the tracings. HSC: Horizontal Semicircular Canal. The black arrows indicate the direction of the endolymphatic flow.

**Figure 3:**
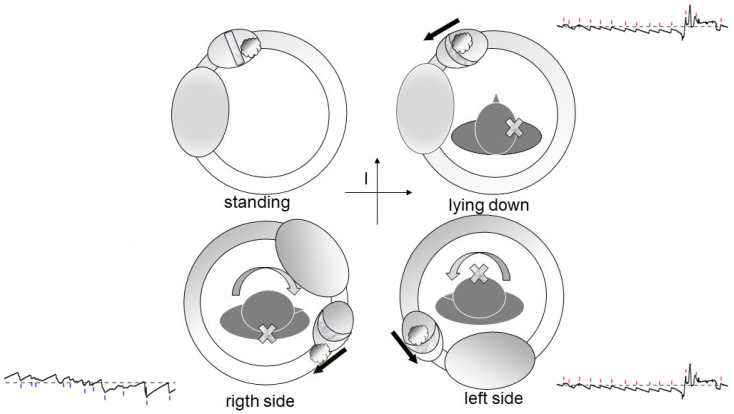
Movement of free-floating otoconia in the horizontal semicircular canal during upright, supine, right-lateral, and left-lateral positions. Gravity-driven shifts in debris produce predictable endolymphatic flow and horizontal nystagmus, illustrated in the corresponding eye-movement recordings. The black arrows indicate the direction of the endolymphatic flow.

**Figure 4:**
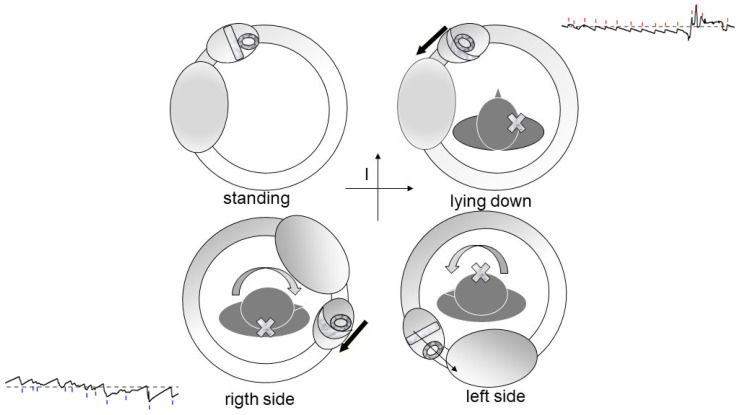
Model of a partial “sieve-type” canal jam in the horizontal canal. Debris permits limited endolymphatic flow, generating monopositional apogeotropic nystagmus when the head is turned toward the side. Lack of flow in the opposite position produces no nystagmus. The black arrows indicate the direction of the endolymphatic flow.

Based on theoretical modeling, we expect:

Debris in Quadrant I [17]: Whether located on the utricular (Figure 2) or canal (Figure 3) side of the ampulla, the outcome is the same—a nystagmus directed toward the affected side upon clinostatism, with apogeotropic bipositional nystagmus. This pattern is also indistinguishable from cupula-adherent debris, regardless of side. The only clinical and putative method to differentiate between a canal-side and utricle-side jam is an ex-juvantibus criterion, whether the liberatory maneuver is effective on the affected or opposite side [18]. A “sieve jam,” in which the obstruction permits only partial endolymphatic flow (Figure 4), produces monopositional apogeotropic nystagmus that appears only when the head is turned toward the healthy side and beats toward the healthy side. When the head is turned to the healthy side, the debris contacts and deflects the cupula; when the head is turned toward the affected side, no pressure gradient is generated due to the position of the debris, and no nystagmus is elicited. An alternative explanation for monopositional apogeotropic nystagmus should also be considered: otoconia may occasionally lie in a canal location where only one of the two head rotations generates a sufficient endolymphatic gradient to induce cupular deflection, while the opposite rotation produces an endolymph movement below the activation threshold.

**Figure 5:**
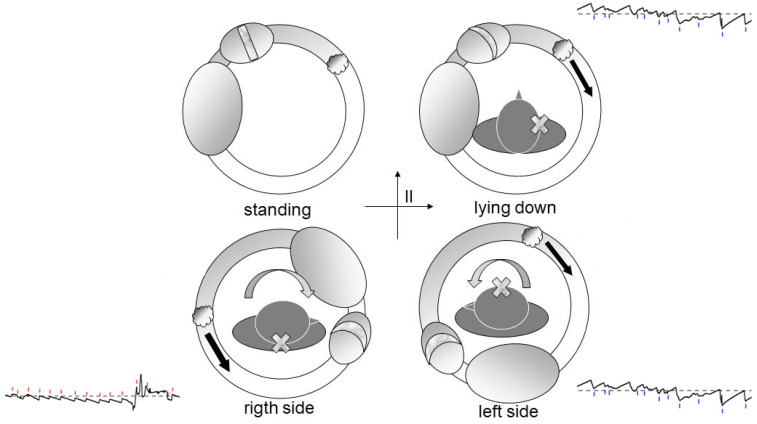
Geotropic variant model showing debris in Quadrant II of the horizontal semicircular canal. The black arrows indicate the direction of the endolymphatic flow.

Debris in Quadrants II or III (Figure 5): These configurations correspond to classic geotropic forms, with supine nystagmus beating toward the healthy ear.

**Figure 6:**
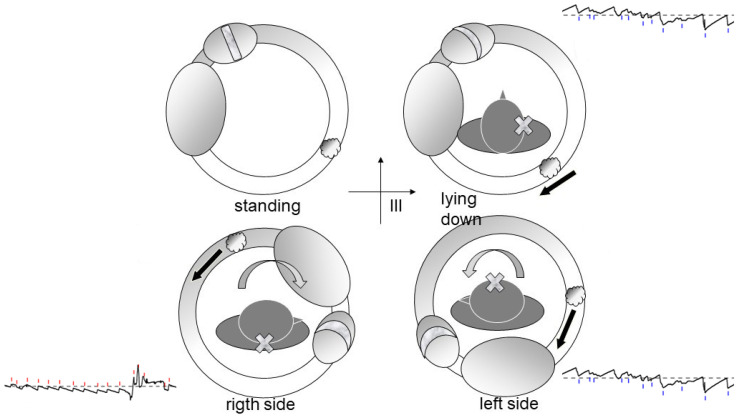
Geotropic variant model showing debris in Quadrant III of the horizontal semicircular canal. The black arrows indicate the direction of the endolymphatic flow.

Debris in Quadrant IV (Figure 6): In contrast, this scenario leads to supine nystagmus beating toward the affected ear yet still results in a geotropic pattern during head turns.

Regarding nystagmus characteristics, particle swarms typically produce paroxysmal nystagmus, whereas conglomerated debris may cause more persistent responses, independent of their location.

The clinical data reveal a noteworthy observation: over half of the cases lacked nystagmus when transitioning to the supine position. While its presence can help localize the lesion, its absence does not rule out canalolithiasis.

Possible explanations include:

1. Debris may initially be too dispersed to generate an effective endolymphatic current but later aggregate into a mass capable of inducing symptoms.
2. A non-mobile jam may not move during the supine transition but only during head rotations.

**Figure 7:**
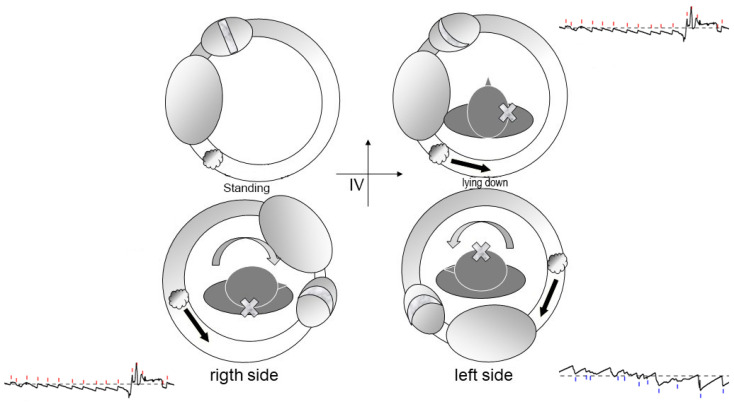
Debris located in Quadrant IV of the horizontal canal generates supine nystagmus beating toward the affected side, while still producing geotropic nystagmus during lateral head rotation. Endolymphatic flow directions and corresponding nystagmus are shown in the tracings. The black arrows indicate the direction of the endolymphatic flow induced by the displacement of the debris.

Excluding cases without lying-down nystagmus, debris was presumed to be located in:

- Quadrant I: 32.2%,
- Quadrants II and III: 56.7%,
- Quadrant IV: 11.1% (Figure 7).

The relative rarity of debris in Quadrant IV may be explained by centrifugal forces acting during natural horizontal head movements, which tend to move debris toward other canal regions.

In 2 of 29 apogeotropic cases (6.9%), monopositional apogeotropic nystagmus suggested a sieve-type jam. These cases require careful differential diagnosis, and the most reliable indicator remains the response to liberatory maneuvers [18].

One apogeotropic case featured “incongruent” supine nystagmus—likely an anecdotal outlier, potentially due to patient head misalignment or canal angle variability during testing.

Our findings confirm the diagnostic relevance of the lying-down nystagmus (LDN) previously described in the literature [7,8,9,10,11,12]. The nystagmus observed upon transitioning to the supine position corresponds to the predicted endolymphatic currents generated by the movement of otoconial debris within the lateral canal [9,10].

The direction of LDN reflects whether the otoconia move ampullipetally or ampullifugally, thereby allowing differentiation between geotropic and apogeotropic forms [9,10].

The model was further expanded by Zhang et al. (2022), who described the coexistence of horizontal and vertical nystagmus components during the roll test, consistent with a multi-vector pattern of endolymphatic flow [11].

Similarly, Liu et al. (2024) observed that LDN may occur together with direction-changing nystagmus, suggesting the presence of multifocal debris or partial canal jams influencing the clinical response [12]. This interpretation is consistent with the clinical description of direction-fixed monopositional apogeotropic horizontal nystagmus, considered evidence of a partial or “sieve-type” canal jam, where otoconial debris transiently obstructs the endolymphatic flow [19].

In addition, Giannoni et al. (2023) emphasized that optimizing patient posture after repositioning maneuvers can facilitate canal clearance, while Lee et al. (2025) demonstrated that the body-roll test can serve as a valuable diagnostic complement when the LDN is subtle or ambiguous [13,14].

## 5. Conclusions

The proposed quadrant-based model is intended as a conceptual framework rather than a fully validated diagnostic or therapeutic algorithm, but it could offer a plausible and clinically coherent theoretical hypothesis for interpreting the findings observed along the horizontal semicircular canal. Our data suggest that lying-down nystagmus (LDN), as described by Koo et al. (2006) and Han et al. (2006), represents a useful indicator of the initial debris position and therefore a valuable lateralizing sign in horizontal canal BPPV [7,8,9,10]. The coexistence of LDN with direction-changing positional responses, as later reported by Liu et al. (2024), further supports the possibility that multifocal debris movement or partial canal jams may influence the clinical presentation [12]. This interpretation is also compatible with the description of direction-fixed monopositional apogeotropic horizontal nystagmus, which can represent a clinical manifestation of a partial or “sieve-type” canal jam in which otoconial debris transiently obstructs endolymphatic flow [19].

Key takeaways include:

(a) To differentiate between utricular- and canal-side jams, the most reliable clinical indicator remains the side on which the liberatory maneuver is effective. Therefore, the maneuver should be performed first on one side, assessed, and—if ineffective—repeated on the opposite side.
(b) The presence of lying-down (supine) nystagmus provides a helpful diagnostic clue which, when interpreted together with the roll-test response, may assist in topographic localization of the debris. However, its absence does not rule out canalolithiasis.
(c) Our findings appear compatible with the mobile jam hypothesis and support the possibility that this mechanism may contribute to both geotropic and apogeotropic variants [9,10,12].

Furthermore, recent therapeutic studies show that optimized postural strategies after repositioning maneuvers and targeted body-roll techniques can enhance canal clearance and reduce recurrence, underscoring the clinical usefulness of integrating LDN analysis into bedside assessment [13].

## Figures and Tables

**Table 1:** GT0 = Geotropic Type, no supine nystagmus, GT+ = Geotropic Type, supine nystagmus toward the healthy side (congruent), GT− = Geotropic Type, supine nystagmus toward the affected side (incongruent).

Type	N	%
GT+	51	30.00
GT−	10	5.88
GT0	80	47.06
T	141	82.94

**Table 2:** AGT0 = Apogeotropic Type, no supine nystagmus, AGT+ = Apogeotropic Type, supine nystagmus toward the affected side (congruent), AGT− = Apogeotropic Type, supine nystagmus toward the healthy side (incongruent), mAGT = Monopositional Apogeotropic Nystagmus (suggestive of “sieve-type” canal jam).

Type	N	%
AGT+	16	9.41
AGT−	1	0.59
AGT0	10	5.88
mAGT	2	1.18
T	29	17.06

## Data Availability

The original contributions presented in this study are included in the article. Further inquiries can be directed to the corresponding author.
